# A pyroptosis-associated gene risk model for predicting the prognosis of triple-negative breast cancer

**DOI:** 10.3389/fonc.2022.890242

**Published:** 2022-10-06

**Authors:** Pengjun Qiu, Qiaonan Guo, Kelun Pan, Jianpeng Chen, Jianqing Lin

**Affiliations:** Department of Breast and Thyroid Surgery, The Second Affiliated Hospital of Fujian Medical University, Quanzhou, China

**Keywords:** pyroptosis, TNBC, immune cell infiltration, risk model, estimate

## Abstract

**Background:**

Pyroptosis is a novel identified form of inflammatory cell death that is important in the development and progression of various diseases, including malignancies. However, the relationship between pyroptosis and triple-negative breast cancer (TNBC) is still unclear. Therefore, we started to investigate the potential prognostic value of pyroptosis-associated genes in TNBC.

**Methods:**

Thirty-three genes associated with pyroptosis were extracted from previous publications, 30 of which were identified in the Molecular Taxonomy of Breast Cancer International Consortium (METABRIC) cohort. On the basis of the 30 pyroptosis-related genes, patients with TNBC were divided into three subtypes through unsupervised cluster analysis. The prognostic value of each pyroptosis-associated gene was assessed, and six genes were selected by univariate and LASSO Cox regression analysis to establish a multigene signature. According to the median value of risk score, patients with TNBC in the training and validation cohorts were separated to high- and low-risk sets. The enrichment analysis was conducted on the differentially expressed genes (DEGs) of the two risk sets using R clusterProfiler package. Moreover, the ESTIMATE score and immune cell infiltration were calculated by the ESTIMATE and CIBERSORT methods. After that, the correlation among pyroptosis-associated risk score and the expression of immune checkpoint-associated genes as well as anti-cancer drugs sensitivities were further analyzed.

**Results:**

In the training and validation cohorts, patients with TNBC in the high-risk set were found in a lower survival rate than those in the low-risk set. Combined with the clinical characteristics, the pyroptosis-related risk score was identified as an independent risk factor for the prognosis of patients with TNBC. The enrichment analysis indicated that the DEGs between the two risk groups were mainly enriched by immune responses and activities. In addition, patients with TNBC in the low-risk set were found to have a higher value of ESTIMATE score and a higher rate of immune cell infiltration. Finally, the expression levels of five genes [programmed cell death protein 1 (PD-1); cytotoxic t-lymphocyte antigen-4 (CTLA4); lymphocyte activation gene 3 (LAG3); T cell immunoreceptor with Ig and ITIM domains (TIGIT)] associated with immune checkpoint inhibitors were identified to be higher in the low-risk sets. The sensitivities of some anti-cancer drugs commonly used in breast cancer were found closely related to the pyroptosis-associated risk model.

**Conclusion:**

The pyproptosis-associated risk model plays a vital role in the tumor immunity of TNBC and can be applied to be a prognostic predictor of patients with TNBC. Our discovery will provide novel insight for TNBC immunotherapies.

## Introduction

Breast cancer (BC) is the most common type of malignancy in women and is the leading cause of mortality associated with cancer in women worldwide (1). As an aggressive type of BC, triple-negative BC (TNBC) is characterized by the absence of estrogen receptor, progesterone receptor, and human epidermal growth factor receptor–2 (2). It was responsible for around 15%–20% of BC cases in the world (3). Despite the boom in tumor treatment strategies, including surgery, chemotherapy, radiotherapy, and targeted therapy, TNBC remains with a high incidence of local recurrence and distant metastases. More recently, anti-tumor immunotherapy is attracting more and more attention in BC. Biomarker-driven therapies and immune checkpoint inhibitors are proving to be promising options for TNBC treatments (4, 5). However, drug resistance in tumors is still a problem that cannot be ignored in TNBC treatment. Hence, it is urgent to investigate reliable novel biomarkers and risk models to predict the prognosis of patients with TNBC and assess the effectiveness of anti-tumor treatment strategies.

Pyroptosis is a newly discovered form of inflammatory cell death that is of great significance in various diseases, including malignant tumors (6). In 1992, pyroptosis was first identified in macrophages infected with Salmonella typhimurium by Zychlinsky and colleagues (7). In eukaryotic cells, 10- to 20-nm pores are rapidly developed in the cell membrane because of pyroptosis, which typically leads to pore-induced membrane lysis. After that, the cytoplasmic components are delivered into the extracellular environment, together with nuclear condensation and cell swelling. Some types of inflammatory cytokines were further released through the pores (6, 8). According to the different stimulating pathways, pyroptosis can be categorized to caspase-1–dependent pyroptosis, caspase-3–dependent pyroptosis, caspase-8–dependent pyroptosis, and caspase-11–dependent pyroptosis (9). Notably, the inflammasome complex is essential for caspase-1 activation and engaged in tumor proliferation, invasion, and metastasis (10, 11). Accumulating evidence has shown that pyroptosis plays a vital role in tumor initiation and treatment. On the one hand, tumorigenesis and progression are closely associated with the signaling molecules and proteins that participated in the pyroptosis, for instance, gasdermins (GSDMs) and inflammasomes. On the other hand, various chemotherapy drugs can trigger pyroptosis in different kinds of tumor cells by means of classical or non-classical inflammasome signaling pathways (6, 12–15).

In 1863, Rudolf Virchow first identified and reported the potential relationship between inflammation and carcinoma. He proposed that chronic inflammation was able to promote the biological processes of inflammatory cells in tumors (16). Recently, some kinds of inflammasomes were identified to be correlated to tumor immune microenvironment (TIME), including nucleotide-binding oligomerization domain–like receptor (NLR) proteins family pyrin domain containing 1 (NLRP1), NLR family pyrin domain containing 3 (NLRP3), and absent in melanoma 2 (AIM2) (11, 17). Ershaid and associates investigated the relationship between pyroptosis and TIME in BC, proposing that immune cell collections could be induced by inflammasome-mediated pyroptosis and interleukin-1β (IL-1β) released to further promote tumor development (18). Another study in head and neck squamous cell cancer indicated that IL-1β could be activated by NLRP3 that is related to the anti-tumor immune responses and tumor progression (19). As a type of pore-forming proteins, GSDM family played a key role in pyroptosis of cancer cells (20). Chemotherapy drugs such as etoposide, 5-fluorouracil (5-FU), and cisplatin could trigger pyroptosis in cancer cells with high expression of GSDME (21). Zhang et al. demonstrated that the caspase-3–dependent GSDME cleavage could be activated by cisplatin and further induce A549 lung cancer cells pyroptosis (22). Moreover, a study in gastric cancer suggested that 5-FU was involved in switching caspase-3–dependent apoptosis into pyroptosis by inducing activation of caspase-3 and cleavage of GSDME instead of GSDMD (21).

Recently, immunologists have been increasingly interested in the role that pyroptosis plays in immune system activation. At the same time, pyroptosis-mediated therapies have made great progresses in anti-tumor treatment strategies, in which cancer cells were eliminated by immune responses (23). Academics suggested a double-edged role of pyroptosis played in the pathogenesis and treatment of carcinoma (24). On the one hand, pyroptosis mediated by inflammasome and cytokines produced by pyroptosis can alter the TIME and help tumor cells escape from immune surveillance. On the other hand, the cytokines released from pyroptosis process can enhance the efficacy of tumor immunotherapies by recruiting immune cells and activating immune responses (23). A previous study in BC revealed that the monocytes in the tumor microenvironment (TME) could be collected by IL-1β and further regulated the tumor cells proliferation (25). In addition, some other research studies indicated that inflammasome NLRP3 could make IL-1β secretion increased and T cell responses suppressed, which were associated with tumor development in melanoma (26). Moreover, inflammatory disease was a precancerous condition of some kinds of malignant tumors. Such chronic inflammation suppressed the anti-tumor immune responses regulated by immune cells including natural killer (NK) cells, M1 macrophages, and T cells (6, 27).

Given the available results, pyroptosis was considered significant in tumor progression and anti-tumor immunotherapy. However, there are still seldom studies to investigate the role of pyroptosis in TNBC. In the current study, bioinformatic analysis was employed to explore the relationship between pyroptosis-related genes and TIME in TNBC. A pyroptosis-associated risk model was established to evaluate the prognosis of patients with TNBC to provide new insights into the combination treatment strategies for TNBC.

## Materials and methods

### Workflow

**Figure 1:**
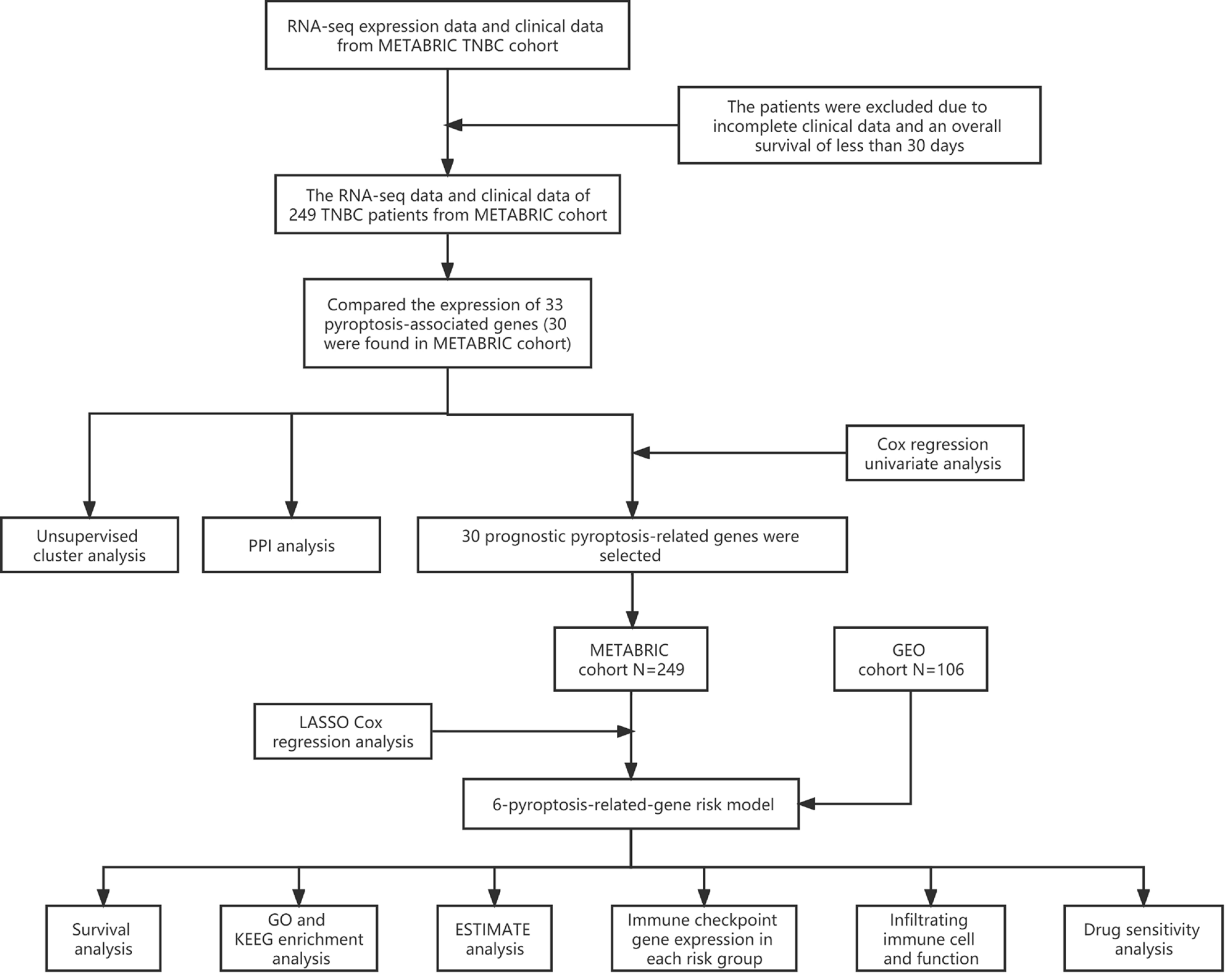
Analysis flow chart.

A combination of multiple bioinformatic approaches was employed to establish a six-pyroptosis-associated gene risk model and investigate the potential mechanisms by which such genes affect the prognostic outcomes of TNBC (**Figure 1**).

### Public data acquisition

The RNA-sequencing (RNA-seq) expression data and clinical characteristics of patients with TNBC were downloaded from METABRIC database in cBioPortal (http://www.cbioportal.org/), and the corresponding information from GSE103091 was extracted from GEO database (https://www.ncbi.nlm.nih.gov/geo/). The inclusion criteria for samples are as follows (1): samples excised from primary tumors; (2) samples have both completed prognostic information and transcriptome expression data. The exclusion criteria for samples are as follows: (1) sample with an overall survival (OS) of less than 30 days; (2) samples with incomplete clinical information. Then, the “limma” package was utilized to determine the normalization of gene expression. As a result, 249 TNBC samples from METABRIC database were enrolled as the training cohort and 106 TNBC samples from GEO database were enrolled as the validation cohort for further study. This study did not involve a human subject trial. Instead, the data came exclusively from the METABRIC and GEO database. The study was conducted in accordance with the Declaration of Helsinki (as revised in 2013). Moreover, 33 genes associated with pyroptosis were extracted from previous publication (28) and presented in **Supplementary Table 1**. The selection criteria for pyroptosis-related genes are as follows: (1) the pyroptosis-related genes identified in TNBC tissues; (2) the average expression level of the gene is greater than 1. Consequently, 30 pyroptosis-related genes identified in TNBC tissues were selected for subsequent research.

### Establishment of protein–protein interaction network

The Search Tool for the Retrieval of Interacting Genes (STRING) database was employed to establish the protein–protein interaction (PPI) network and conduct the correlation analysis. The PPI pairs were selected with a combination score of 0.4 and visualized using Cytoscape. The degree of each node in the PPI network was further calculated, and the Pearson correlation analysis of pyroptosis-related genes was conducted by R software (version 4.0.5).

### Unsupervised cluster analysis

According to the expression levels of pyroptosis-related genes, unsupervised clustering analysis was performed to distinguish disparate patterns of pyroptosis and to categorize the patients with TNBC in the training cohort (METABRIC cohort) for a subsequent study. The unsupervised clustering analysis was conducted using the “ConsensuClusterPlus” R package with 1,000 times repetitions. Moreover, the consensus clustering algorithm was applied to confirm the optimal number of clusters.

### Construction of the pyroptosis-associated risk model

The pyroptosis-associated independent prognostic genes were selected by Cox regression univariate analysis of OS and visualized with forest plots. Subsequently, the least absolute shrinkage and selection operator (LASSO) Cox regression model was established to minimize redundant genes and avoid overfitting of the model. As a consequence, final independent prognostic pyroptosis-related genes were screened out by this model. The expression levels of independent prognostic pyroptosis-associated genes were applied to establish the following formula: Risk score = $\sum_{i=1}^{n}\left(Exp_{i}*Coe_{i}\right)$ (N = 6, *Exp*_*i*_ denoted the expression level of each gene picked up, and *Coe*_*i*_ represented the consequential Cox regression coefficient). After that, patients with TNBC in the training and validation cohorts were respectively divided into low- and high-risk groups according to the median risk score of the training cohort. On the basis of the gene expression signature, the “prcomp” function of the “stats” R package was employed to perform the principal component analysis (PCA). Subsequently, the survival analysis was conducted between high- and low-risk groups using the R package “survminer”. Time-dependent receiver operator characteristic curve (ROC curve) analysis was also carried out to assess the predictive efficacy of this risk model. Moreover, univariate and multivariate COX regression analyses were performed on the training cohort to identify the independent prognostic factor for patients with TNBC.

### Enrichment analysis

The differentially expressed genes (DEGs) between high- and low-risk groups in the METABRIC and GEO cohorts were identified by the “edgeR” package and “limma” package, respectively [value fold changes (FC) with |logFC| >1 and adjusted P-value<0.05 as cutoff values]. The DEGs of METABRIC cohort were presented in **Supplementary Table 2** and those of GEO cohort were presented in **Supplementary Table 3**. Gene oncology (GO) and Kyoto Encyclopedia of Genes and Genomes (KEGG) enrichment analyses were performed for DEGs between different risk groups by the R package “clusterProfiler”. P-values<0.05 were considered statistically significant in enrichment analysis.

### Assessment of the tumor immune microenvironment and immune cell type components

Estimation of Stromal and Immune cells in Malignant Tumor tissues using Expression (ESTIMATE) algorithm is consist of stromal score, immune score, and ESTIMATE score. The ratio of the immune-stromal component of TME was calculated by the “estimate” package in R software and represented as respective score. Subsequently, CIBERSORT (http://cibersort.stanford.edu/) was applied to estimate and analyze the immune cell infiltration through RNA-seq expression profiles. LM22 was employed to show the annotated gene expression signatures of 22 marked immune cell subtypes, including T-cell subsets, naive and memory B cells, plasma cells, and myeloid subsets. The assumption of immune cell types was considered accurate and statistically significant for subsequent analysis with the P< 0.05. Moreover, the fractions of tumor immune-infiltrating cell (TIIC) types for each tumor sample were calculated using the CIBERSORT algorithm. The Wilcoxon test was performed to distinguish the features of TIIC among different risk set tissues.

### The relationship among immune checkpoint genes and pyroptosis-associated risk score

Five genes associated with immune checkpoint were extracted from previous publications. The correlation between the expression levels of immune checkpoint genes and pyroptosis-associated risk score was identified by the R package “GGPUBR”, “ggplot2”, and “ggExtra”. P< 0.05 was considered statistically significant.

### Drug sensitivity analysis

The mRNA profiles and drug sensitivity half-maximal inhibitory concentration (IC50) values of the NCI-60 panel of human cancer cell lines were downloaded from CELLMINER website (https://discover.nci.nih.gov/cellminer/). After that, the therapeutic efficacies of 163 Food and Drug Administration–approved drugs were confirmed in patients suffering from TNBC. The significance of differences in IC50 Z-score among different risk groups was assessed by the Wilcoxon test and visualized by R package “ggplot2”.

### Statistical analysis

All statistical analysis were conducted by R software (version 4.0.5) (https://www.r-project.org/). The Wilcoxon test was applied to assess the differences of variables in two risk groups. The Kaplan–Meier curve was employed to analyze the survival data. Pearson correlation analysis was applied to identify the relevance between the degree of each node in the PPI network and pyroptosis-related genes. P< 0.05 was considered statistical significance.

## Results

### Interactions among pyroptosis-associated genes

**Figure 2:**
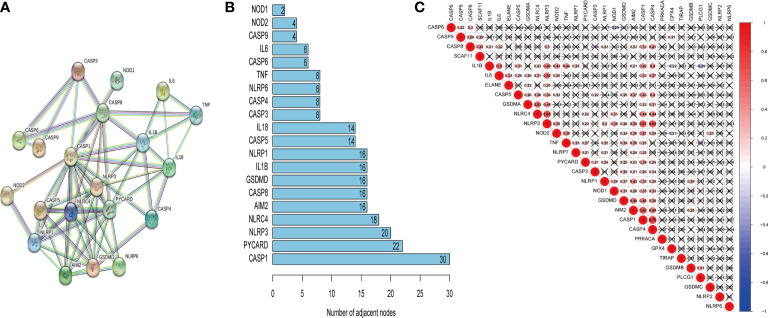
The correlation of pyroptosis-related genes. The PPI network of pyroptosis-related genes **(A)**, number of interaction nodes among pyroptosis-related genes **(B)**, and Pearson correlation analysis of pyroptosis-related genes **(C)**. Red indicates positive correlations, and blue indicates negative correlations. The darker the color, the stronger the correlation. X indicates p > 0.05.

The PPI network was developed to illustrate the relationship between pyroptosis-related genes (**Figure 2A**). The STRING database was adopted to analyze the interactions among selected genes, indicating that CASP1 was a hub gene of the pyroptosis-associated genes and interacted with 30 nodes (**Figure 2B**). Subsequently, further analysis suggested a strong positive correlation between CASP1 and other pyroptosis-related genes, especially CASP4 (**Figure 2C**). Moreover, CASP1 was also positively related with AIM2 and NLRP3.

### Tumor classification based on pyroptosis-related genes

**Figure 3:**
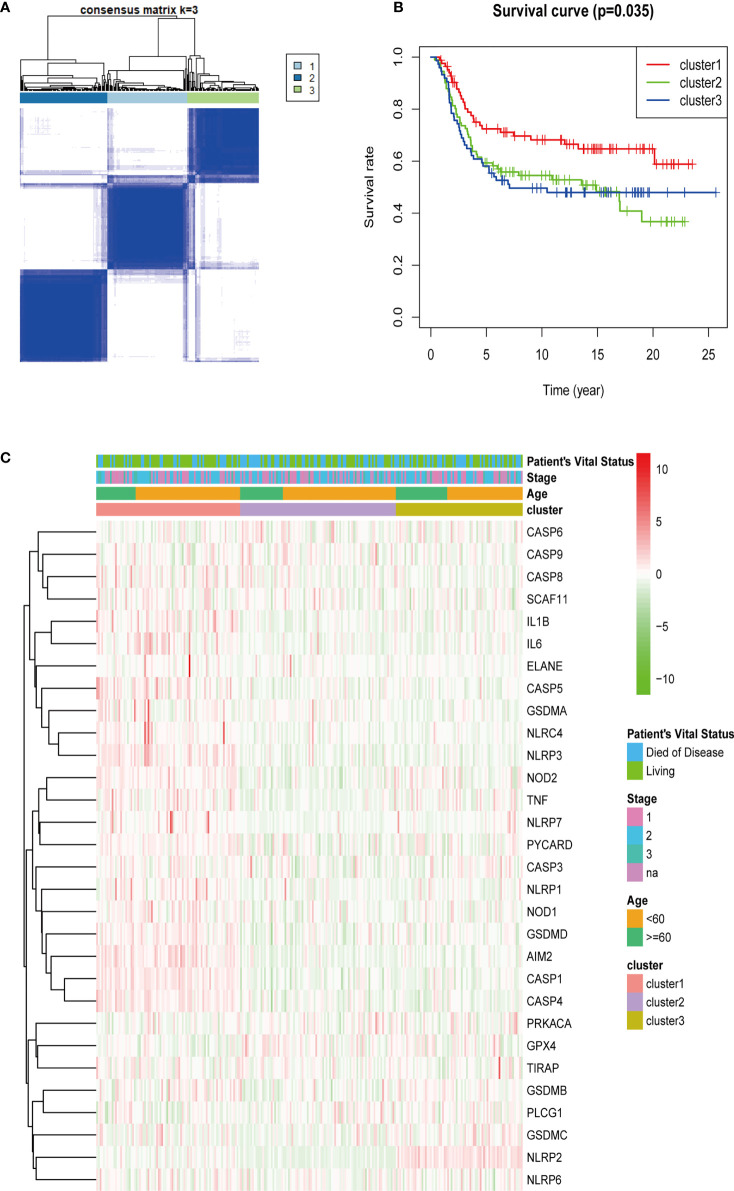
Tumor classification based on pyroptosis-related genes. A total of 249 patients with TNBC were grouped into three clusters according to the consensus clustering matrix (k = 3) **(A)**. Kaplan–Meier over survival curves for the three clusters **(B)**. Heatmap and the clinicopathologic characters of the three clusters classified through these DEGs **(C)** (stage, AJCC tumor stage I/II/III/NA).

Thirty genes related to pyroptosis in the METABRIC cohort were selected. The unsupervised cluster analysis was performed with 249 patients with TNBC in the METABRIC cohort to investigate the relationships between the expression levels of 30 pyroptosis-related genes and TNBC subtypes. The clustering variable (k) was increased from 2 to 10 to select the best one meeting the condition that the intergroup correlations were lowest and that the intragroup correlations were the highest. Hence, on the basis of the 30 pyroptosis-related genes, 249 patients with TNBC were divided into three clusters (k = 3) (**Figure 3A**). Subsequently, the survival analysis was conducted for each cluster, and the result was considered statistically significant with P = 0.035 (**Figure 3B**). After that, the heatmap was drawn to explore the differences of gene expression levels and clinical characteristics (age, <60/≥60 years; tumor stage, AJCC I/II/III/NA; vital status of patients, died/living) among the three clusters (**Figure 3C**).

### Construction of pyroptosis-related risk model to predict the prognosis of patients with TNBC

**Figure 4:**
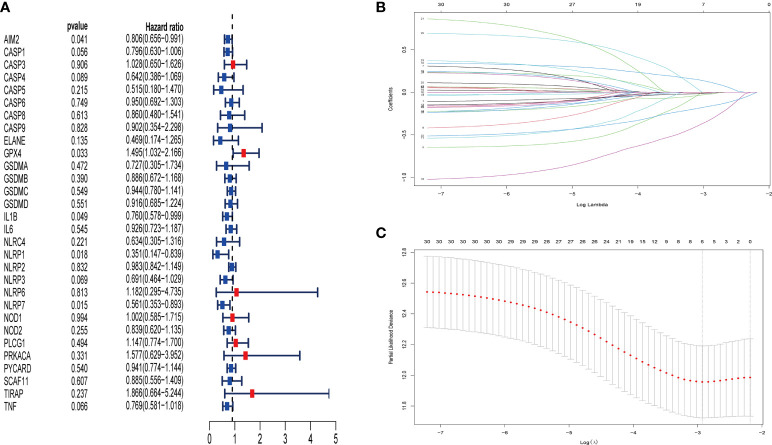
Identification of pyroptosis-associated genes in patients with TNBC with prognostic significance by univariate Cox regression analysis and LASSO Cox regression analysis. Thirty pyroptosis-associated genes were identified to be associated with prognosis in patients with TNBC *via* univariate Cox regression analysis (red point indicates HR value > 1, blue point indicates HR value< 1) **(A)**. LASSO coefficient profiles of 30 pyroptosis-associated genes with P< 0.01 **(B)**. The results of the fivefold cross-validation determined the optimal value of the penalty parameter λ (λ=0.05346444). Six independent prognostic genes for risk model construction were identified **(C)**.

For the purposes of building the pyroptosis-related prognostic signature, univariate Cox regression analysis was applied to initial selecting on 30 overlapping genes in the METABRIC database. As a result, the excess confounding genes were eliminated, and the genes with the greatest prognostic impact were screened out. The relevance of each gene and OS was presented *via* forest plot (**Figure 4A**). After that, LASSO regression analysis was employed to prevent the elimination of vital genes associated with prognosis and to identify six independent prognostic genes for risk model construction. The LASSO coefficient overview of the selected genes was presented in **Figure 4B**, and fivefold cross-validation outcomes were generated to determine the preferred value of the penalty parameter λ (λ = 0.05346444) (**Figure 4C**). The six finally selected genes were AIM2, GPX4, IL1B, NLRP1, NLRP3, and NLRP7. Subsequently, the signature to assess the prognosis of patients with TNBC was developed according to the expression levels of the finally selected genes and the regression coefficients as described below: Risk score = (−0.012 × expression level of AIM2) + (0.122 × expression level of GPX4) + (−0.026 × expression level of IL1B) + (−0.337 × expression level of NLRP1) + (−0.004 × expression level of NLRP3) + (−0.168 × expression level of NLRP7). Accordingly, patients in the METABRIC training cohort were divided into high- and low-risk groups based on the median risk score. Furthermore, the risk scores of 106 samples of GSE103091 were calculated on the basis of the risk model, respectively. Similarly, the patients in the validation cohort were separated into high- and low-risk groups according to the cutoff value of the training cohort risk score.

**Figure 5:**
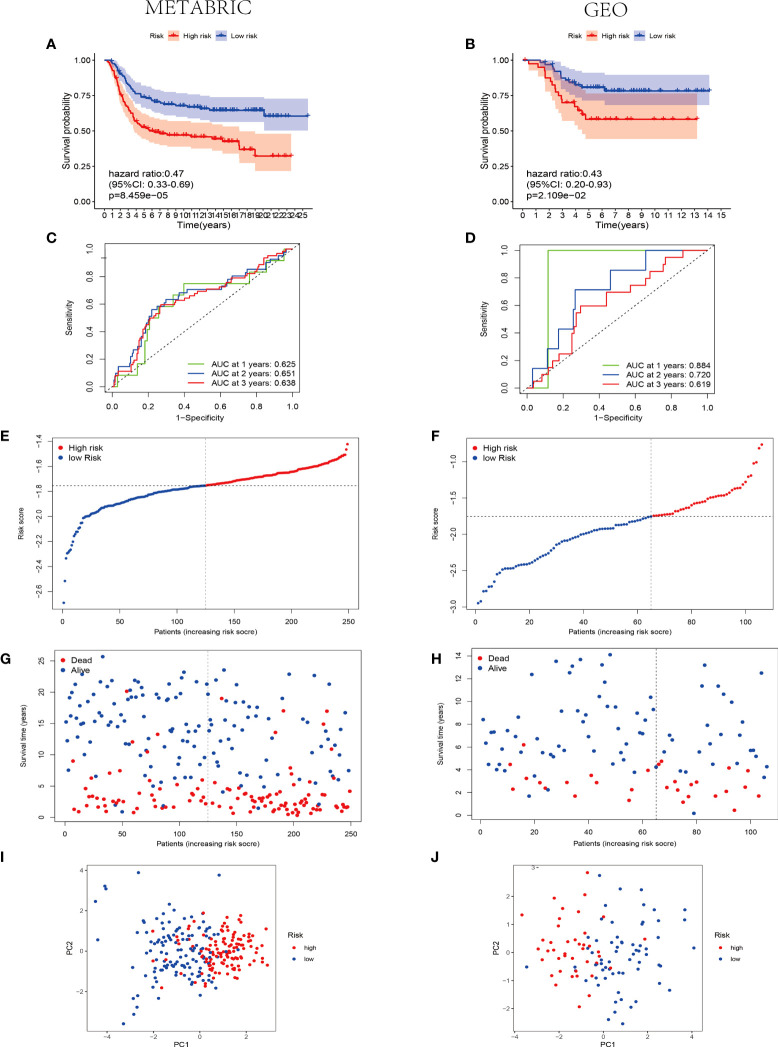
Efficacy and prognosis analysis of the pyroptosis-associated signature of patients with TNBC. **(A, B)** Kaplan–Meier survival curves for patients with TNBC from the METABRIC **(A)** and GEO groups **(B)**, stratified on the basis of risk scores (high vs. low); the hazard ratio of the METABRIC and GEO cohorts are 0.47 and 0.43, respectively (95% CI = 0.33–0.69 for the METABRIC cohort and 0.20–0.93 for the GEO cohort); comparisons of the survival time in high- and low-risk groups with log-rank tests (P = 8.459E-05 and P = 2.109E-02, respectively). **(C, D)** ROC curve analysis of model accuracy for predicting the prognosis of patients with TNBC at 1, 2, and 3 years in the METABRIC **(C)** and GEO **(D)** groups. **(E, F)** The median value and distribution of the risk score in the METABRIC **(E)** and GEO **(F)** groups. **(G, H)** The distributions of survival status and risk scores in the METABRIC **(G)** and GEO **(H)** groups. **(I, J)** PCA analysis plot of the METABRIC **(I)** and GEO groups **(J)**. Red point indicates patients with high risk, and blue point indicates patients with low risk.

As shown in **Figures 5A, B**, patients with TNBC with low-risk score were suggested better survival rates by the Kaplan–Meier curves in the training and validation sets (P< 0.01). Subsequently, the time-dependent ROC analysis was applied to evaluate the predictability of efficacy of the signature at 1, 2, and 3 years. Consequently, the prognostic signature was validated robustly efficient in forecasting the OS of patients with TNBC through the area under the curve (AUC) (AUC = 0.625, 0.651, and 0.638 at 1, 2, and 3 years in the training set; AUC = 0.884, 0.720, and 0.619 at 1, 2, and 3 years in the validation set; **Figures 5C, D**). Hence, the six-pyroptosis-associated gene risk model to evaluate the prognostic outcomes of patients with TNBC was created, and the patients in the training cohort and the validation cohort were divided into different risk groups according to the risk scores (**Figures 5E, F**). As suggested by **Figures 5G, H**, the patients with TNBC with lower risk scores were manifested better prognostic outcomes rather than those with higher risk scores. PCA was conducted to both the training and validation cohorts, resulting that the patients in distinct risk groups were categorized in the opposed directions (**Figures 5I, J**).

Moreover, the univariate and multivariate Cox regression analysis were conducted to the six-pyroptosis-related gene risk model and other covariates involving in age and stage to identify the independent prognostic factors for patients with TNBC. The univariate Cox regression analysis manifested that the tumor stage and pyroptosis-associated risk score were independent prognostic variables for patients with TNBC in the training cohort (P< 0.001, HR = 2.670, 95% CI = 1.562–4.562 and P = 0.001, HR = 11.734, 95% CI = 2.599–52.988; **Supplementary Figure 1A**). The multivariate Cox regression analysis revealed that the tumor stage and pyroptosis-associated risk score–independent factors for the prognosis prediction of patients with TNBC (P = 0.001, HR = 2.428, 95% CI = 1.420–4.154 and P = 0.003, HR = 10.395, 95% CI = 2.224–48.588; **Supplementary Figure 1B**).

### Functional enrichment analysis

**Figure 6:**
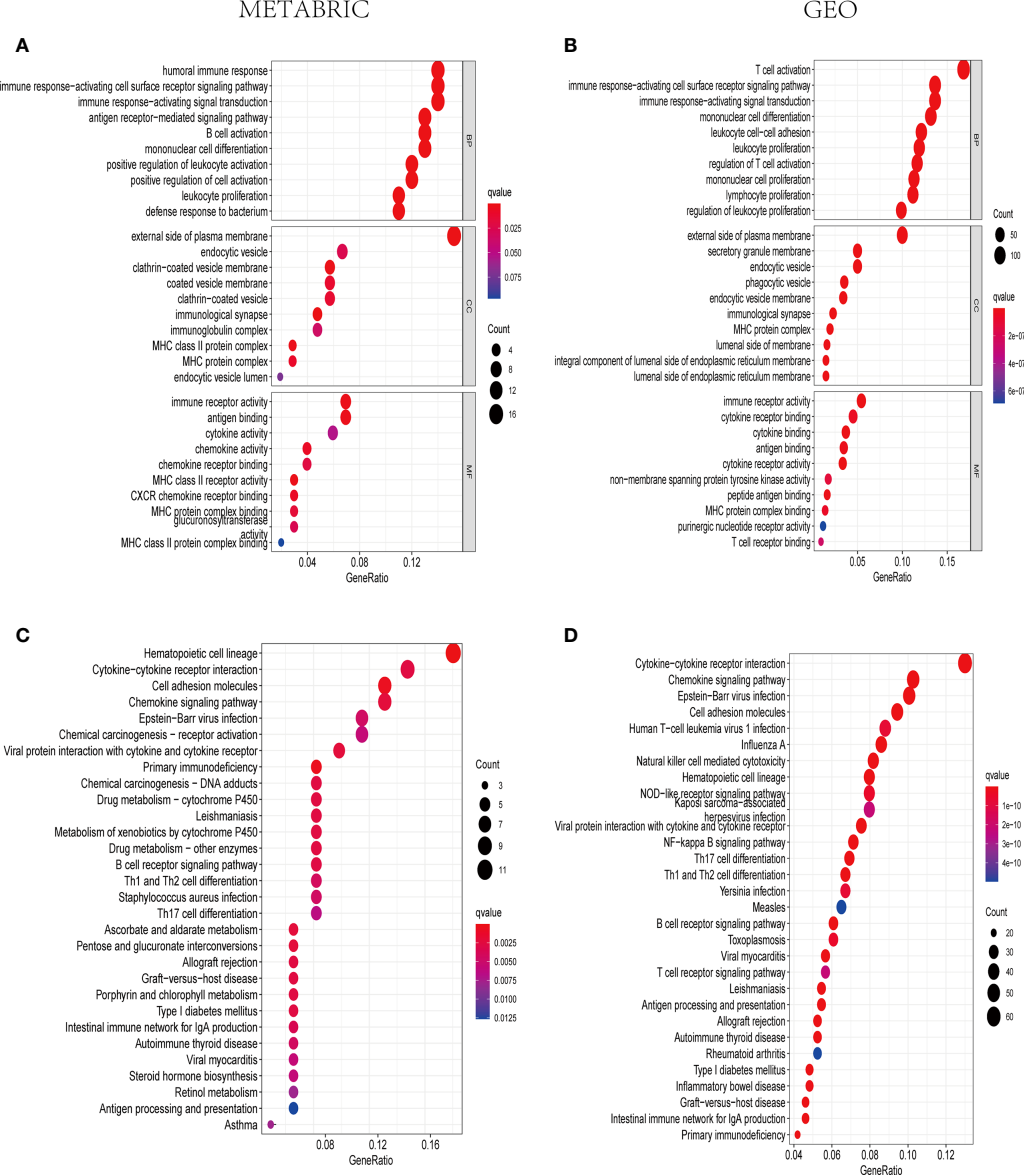
The representative outcomes of GO and KEGG enrichment analysis. The top 10 GO terms of BP, CC, and MF enrichment in DEGs between the high- and low-risk sets in the METABRIC **(A)** and GEO groups **(B)**. The 30 enriched KEGG pathways in DEGs between the high- and low-risk sets in the METABRIC **(C)** and GEO groups **(D)**.

KEGG and GO analyses were applied to the DEGs among high-risk and low-risk groups to find the signaling pathways and biological functions related to pyroptosis-associated risk model. As a consequence, the top 30 GO terms of the training and validation cohorts were shown in **Figures 6A, B**. Moreover, the 30 enriched KEGG pathways in both cohorts were presented in **Figures 6C, D**. A majority of these GO and KEGG outcomes were correlated with immune cell activities and immune response.

### The relevance of the ESTIMATE score and pyroptosis-associated risk model

The ESTIMATE fraction for each patient was calculated through the ESTIMATE algorithm to further manifest the overall extent of immune infiltration. In the training cohort, the stromal score, immune score, and ESTIMATE score were indicated lower in the high-risk group (P< 0.05) (**Supplementary Figures 2A–C**). Analogously, the results in the validation cohort were presented in **Supplementary Figures 2D–F**. Accordingly, the low values of stromal, immune, and ESTIMATE scores were correlated with a poor OS, whereas the high scores were related to a better OS.

### The distribution of infiltrating immune cells in TNBC

**Figure 7:**
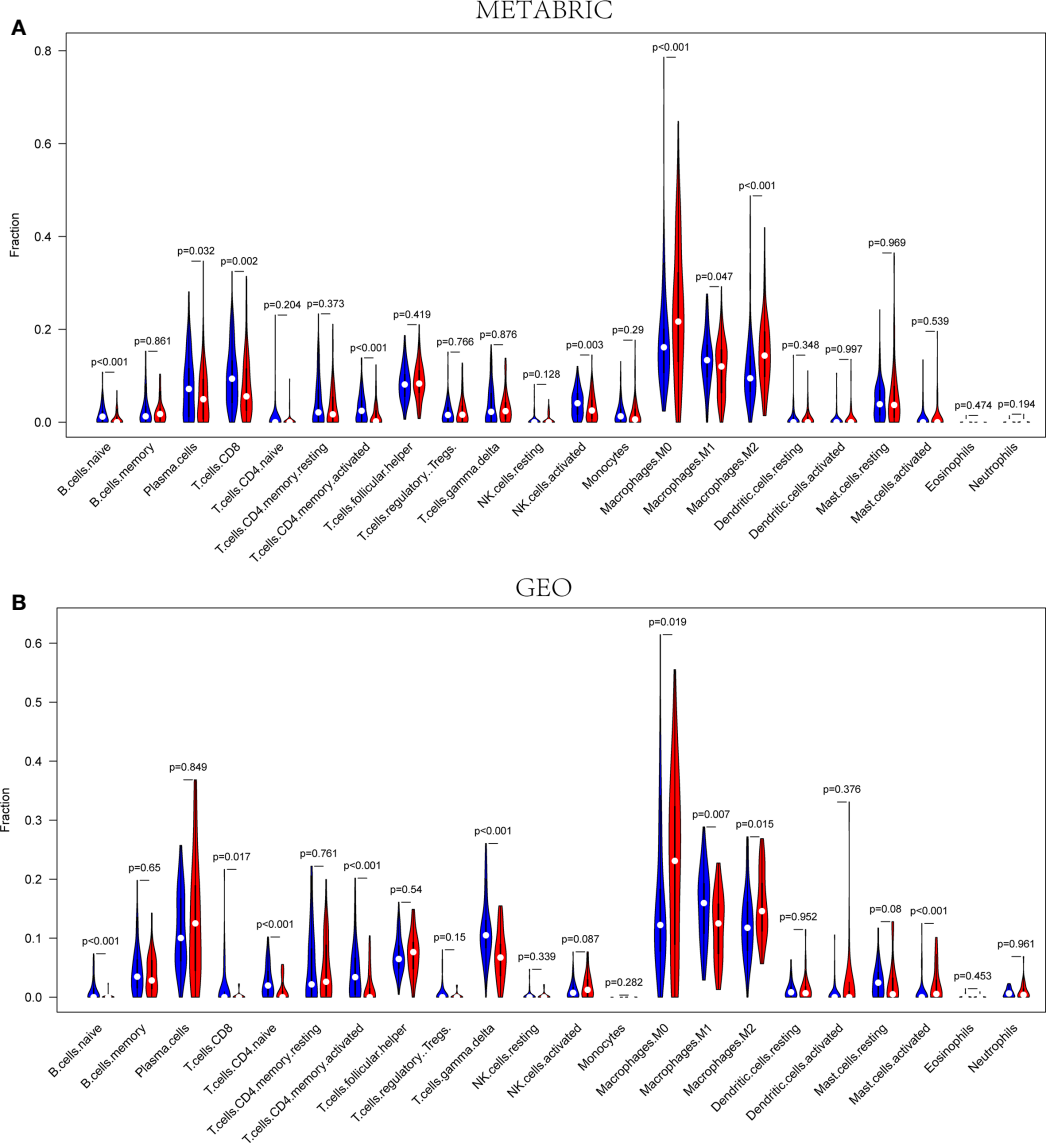
The infiltration fractions of different immune cells between the high- and low-risk groups in the METABRIC **(A)** and GEO **(B)** sets, respectively. Red represents high-risk groups, and blue represents the low-risk group (P< 0.05).

According to the outcomes of GO and KEGG analysis, the DEGs were commonly enriched in the biological functions associated with immune cell activities. Consequently, the CIBERSORT algorithm was applied to TIIC proportion calculation and TIIC profile creation. The infiltration of immune cells in the METABRIC and Gene Expression Omnibus (GEO) cohorts were presented in **Supplementary Figure 3**. As shown in **Figure 7A**, naive B cells (P< 0.001), plasma cells (P = 0.032), CD8+ T cells (P = 0.002), CD4+ activated memory T cells (P< 0.001), activated NK cells (P = 0.003), and M1 macrophages (P = 0.047) were downregulated, whereas M0 macrophages (P< 0.001) and M2 macrophages (P< 0.001) were upregulated in the high-risk group in the METABRIC cohort. As shown in **Figure 7B**, naive B cells (P< 0.001), CD8+ T cells (P = 0.017), naïve CD4+ T cells (P< 0.001), CD4+ activated memory T cells (P< 0.001), gamma-delta T cells (P< 0.001), and M1 macrophages (P = 0.007) were downregulated, whereas M0 macrophages (P = 0.019), M2 macrophages (P = 0.015), and activated mast cells (P< 0.001) were upregulated in the high-risk group in the GEO cohort. Therefore, the studies about pyroptosis-associated genes were able to provide special insight for immunotherapy strategies in patients with TNBC.

### The relevance of immune checkpoint genes and pyroptosis-related risk score

**Figure 8:**
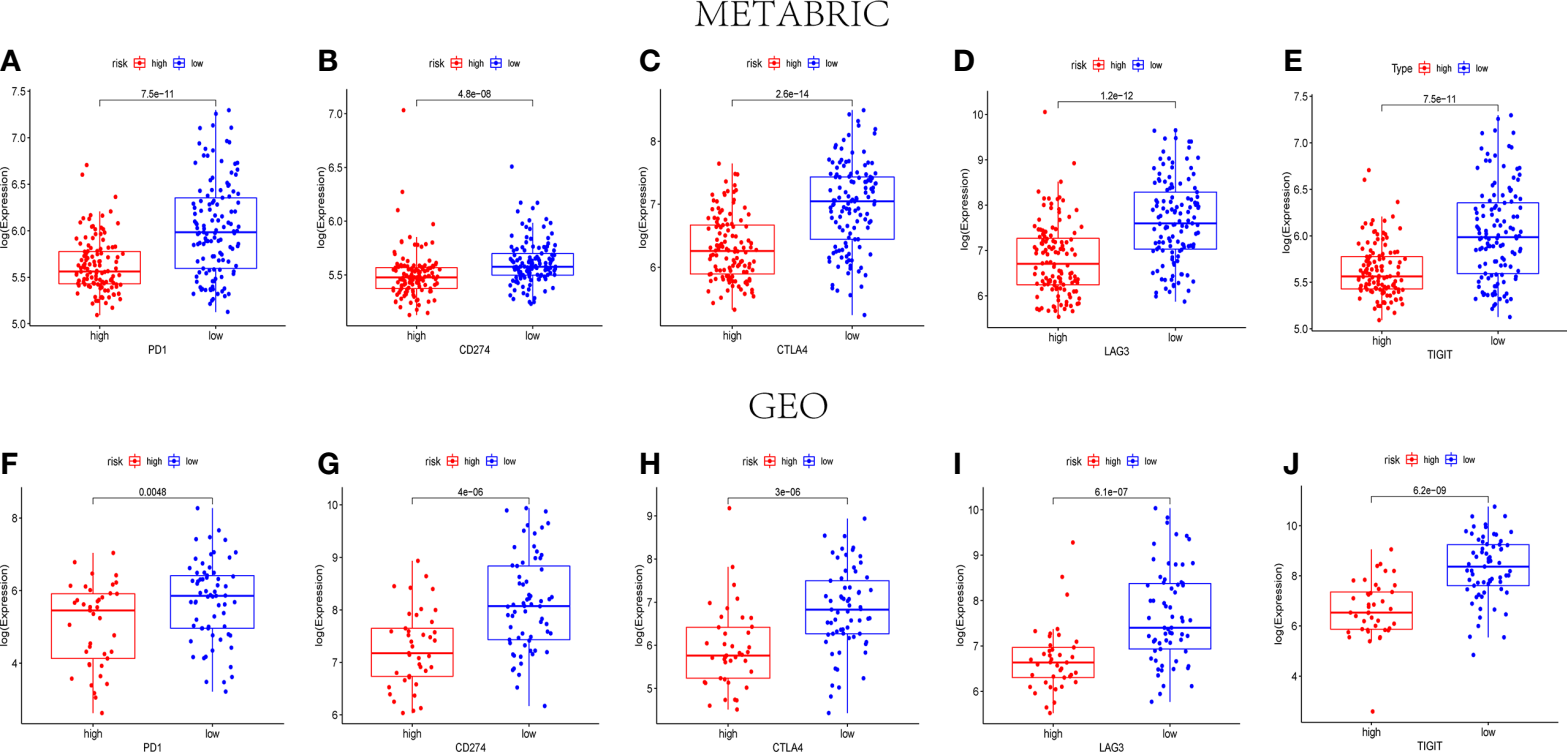
The expression levels of immune checkpoint–related genes between the high- and low-risk groups. The expression levels of PD1 **(A)**, CD274 **(B)**, CTLA4 **(C)**, LAG3 **(D)**, and TIGIT **(E)** in different risk groups of the METABRIC set (P< 0.05). The expression levels of PD1 **(F)**, CD274 **(G)**, CTLA4 **(H)**, LAG3 **(I)**, and TIGIT **(J)** in different risk groups of the GEO set (P< 0.05).

According to the previous reports, PD1, PD-L1(CD274), CTLA4, LAG3, and TIGIT were associated with the targets of immune checkpoint inhibitors. Consequently, their expression levels were detected to identify the relationship between immune checkpoint genes and the pyroptosis-related risk scores. Both in the METABRIC cohort and the GEO cohort, the expression levels of the five genes were lower in the high-risk groups than those in the low-risk groups (P< 0.05) (**Figure 8**).

### Drug sensitivity analysis for pyroptosis-related risk score in TNBC

To obtain the key to precision treatment, the significant differences on drug sensitivity between the low-risk group and the high-risk group were assessed using the CellMiner database. The drug sensitivity was measured by Z-score, and the higher the score, the higher sensitivity to the corresponding drug treatment. The pyroptosis-associated risk score of NCI60 cell lines was obtained, and the relevance of the risk score and the inhibitory centration (IC50) value of six common drugs used in BC were investigated. As a result, Paclitaxel, Vinorelbine, Tamoxifen, Lapatinib, Epirubicin, and Fulvestrant were identified to be significantly related to the pyroptosis-associated signature (|Pearson correlation| > 0.25 and P< 0.05) (**Supplementary Figure 4**). Remarkably, a high-risk score was correlated with a higher half IC50 of Paclitaxel (Cor = 0.433, P< 0.001), Vinorelbine (Cor = 0.421, P< 0.001), Tamoxifen (Cor = 0.421, P< 0.001), Epirubicin (Cor = 0.326, P = 0.011), and Fulvestrant (Cor = 0.287, P = 0.026). Nevertheless, a high-risk score was associated with a lower half IC50 of Lapatinib (Cor = 0.353, P = 0.006). Consequently, the pyroptosis-related risk model was considered as a potential predictor for drug sensitivity in TNBC.

## Discussion

TNBC is a kind of highly heterogeneous tumor with high incidence of distance metastasis and local recurrence (29). Although, there are so many treatments for TNBC, including surgery, chemotherapy, targeted treatment, immunotherapy, and radiotherapy, the prognostic outcomes of patients with TNBC remain unsatisfactory. Hence, it is significant to understand the mechanisms of TNBC progression and find potential biomarkers and targets for effective treatment strategy development. In the current study, PPI network was first constructed on the basis of the 33 previously reported pyroptosis-related genes, 30 of them found in TNBC tissues from the METABRIC database were selected for subsequent analysis. Furthermore, on the basis of the 30 pyroptosis-associated genes, the unsupervised cluster analysis was conducted with 249 patients with TNBC from the METABRIC cohort and separated them into three clusters. The differences of the survival analysis of three clusters were considered statistically significant. After that, a six-pyroptosis-associated gene risk model was established by Cox univariate analysis and LASSO Cox regression analysis to evaluate the prognostic value of six selected genes. Subsequently, a functional analysis manifested that the DEGs between different risk groups were enriched in the pathways related to immune responses. As for the TIME of TNBC, some kinds of immune cells were found upregulated in the low-risk group and the expression of some immune checkpoint genes were also upregulated in the low-risk group. Eventually, the drug sensitivity of some common drugs for TNBC treatment was analyzed on the basis of the pyroptosis-related risk score.

As a newly discovered form of programmed cell death, pyroptosis was reported to serve an important role in the tumor progression and drug resistance in recent years (30). Some normal cells transformed into tumor cells by stimulation of several inflammatory elements secreted from pyroptosis (23, 24). However, promoting the pyroptosis of tumor cells could enhance the sensitivity of tumor cells to drugs. It was reported that tamoxifen inhibited tumor progression through tumor cell pyroptosis induction (31). Moreover, pyroptosis was identified to provide the chance for relieving immunosuppression and enhancing immune responses in BC treatment (32). Hence, the tumor cell pyroptosis promotion could be a promising target for TNBC treatment. In TNBC, the potential mechanisms of the pyroptosis-associated gene interaction and the relationship between the genes and clinical outcomes remain unclear. In this study, a six-pyroptosis-associated gene risk model was established. The six prognostic pyroptosis-related genes were AIM2, GPX4, IL1B, NLRP1, NLRP3, and NLRP7. AIM2 was originally discovered in melanoma, in which its expression level was decreased (28, 33). AIM2 presented different functions in different kinds of malignant tumors. In some cancers, AIM2 was considered as the tumor suppressor, because it was discovered inactivated and mutated in these cancers, for instance, colorectal and gastric cancers (34). However, in non–small cell lung cancer and nasopharyngeal cancer, it was found overexpressed and associated with increased survival of patients (35). Chen and colleagues found that AIM2 could suppressed human BC proliferation in mammary tumor growth in a mouse model (36). Moreover, a study that involved the mechanism of dihydroartemisinin (DHA) inhibiting tumorigenesis in BC indicated that DHA could induce pyroptosis to suppress tumor progression through promoting the AIM2/caspase-3/DFNA5 axis (37). Interestingly, AIM2 seemed to be a protection element for TNBC in our study. AIM2 expression patterns vary from one carcinoma to another, which meant it played a special role in different kinds of tumors. AIM2 was reported to have anti-tumorigenic and carcinogenic effects (6, 38, 39). Given the conflicting outcomes of AIM2 in diverse tumors and limited data about TNBC, our results will provide new insights for further studying the relationship between AIM2 and TNBC. In 1982, glutathione peroxidase 4 (GPX4) was initially identified as a member of the selenium‐dependent peroxidase family (40). A previous study in TNBC to investigate the role of GPX4 played in ferroptosis and apoptosis indicated that GPX4 was remarkably increased in TNBC tissue other than non-TNBC tissue and associated with tumor stages (41). Zhang et al. found that GPX4 was upregulated in pan-cancer and negatively related to prognosis of patients. In addition, the expression of GPX4 was reported associated with chemoresistance of anti-cancer drugs, including topotecan and lapatinib (42). In our study, GPX4 seemed to be a risk gene for TNBC associated with poor prognosis, but the correlation between GPX4 and pyroptosis in TNBC was still unclear. As for interleukin 1B (IL1B), it was reported that IL1B expressed by BC cells made them more aggressive and played an important function in the initiation of the metastatic process (43). Furthermore, previously reported data indicated that abnormal IL1B induction was related to poor prognostic outcomes in most malignant tumor types, including lung cancer, colon cancer, and BC (44). You et al. found that IL1B mRNA expression levels were observed to be higher in TNBC cells compared with that in non-TNBC cells. Moreover, the elevation of IL1B enhanced the aggressiveness of TNBC cells through the production of IL8 and MMPs (45). Interestingly, IL1B was identified as a protected factor to consist of the pyroptosis-related gene signature in our study. There are seldom studies to investigate the mechanisms underlying pyroptosis and IL1B in TNBC. Hence, it is important to conduct more wet experiments to detect the potential regulatory mechanisms. NLRP1 was the first member of NLR family protein that could form inflammatory vesicles with the ability to activate caspases and induce cellular inflammatory response (46–48). Some academics suggested that NLRP1 could promote melanoma proliferation through inflammatory activation enhancing, which was associated with apoptosis (49). Moreover, Wei and colleagues demonstrated that the overexpression of NLRP1 was related to the tumorigenesis of BC. It promoted the growth, migration, and invasion of BC cells by inducing EMT (48). However, the study in lung adenocarcinoma conducted by Edward et al. confirmed that the decreased expression of NLRP1 was associated with a low ratio of immune cell infiltration and poor prognostic outcomes (50). NLRP3 was another member of the NLR family that played a protective role in multiple cancers. The capability of NLRP3 to response to various signals contributed to its biological significance in many diseases, including transplantable tumors, melanoma, and colorectal cancer (51). A previous study in metastasized colonic tumor indicated that IL18 secretion mediated by NLRP3 was able to induce tumoricidal activities of NK cells against metastatic colon cancer cells in the liver of mouse (52). Moreover, the NLRP3 inflammasome was also essential for the adaptive immune responses to anti-cancer (51). There are seldom studies involved in the correlation between NLRP7 inflammasome and malignant tumors. In a study on gestational choriocarcinoma (CC), NLRP7 was categorized as one of the key players in the development of CC. The results derived from the study by Reynaud et al. demonstrated that NLRP7 was involved in the proliferation of CC cells directly and suppressed the maternal immune response to further promote tumor development and migration (53). Nevertheless, the relationship between the expression of NLRP7 and the prognosis of patients suffering from malignant tumors were still unclear. According to our results, NLRP7 played a protective function in the six-pyroptosis-related gene signature and associated with a better prognosis of patients with TNBC. It is important to conduct more studies to investigate the underlying relationship among NLR family and the prognosis of patients with TNBC.

To date, pyroptosis has not been sufficiently investigated, despite some identified resemblances to apoptosis and some mechanisms of crossover. With the development of tumors, several modes of cell death are likely to coexist and mutually interact. Recently, it was reported that pyroptosis in normal cells probably modified the microenvironment and sped the immune evasion, thus giving protection against tumorigenesis (54). In addition, multiple treatment therapies were found to activate the immune system as pyroptosis was induced in tumor cells (23). In our study, the DEGs between different risk sets were included in the GO and KEGG enrichment analyses, indicating that immune cell activation and immune responses were closely related to the DEGs. According to the outcomes of our enrichment analyses, a reasonable speculation can be made that pyroptosis can modulate the TIME composition. As shown in **Figure 7**, naive B cells, CD8+ T cells, CD4+ activated memory T cells, and M1 macrophages were downregulated, whereas M0 macrophages and M2 macrophages were upregulated in the high-risk groups in the METABRIC cohort, and theses outcomes were verified in the GEO cohort. Pyroptosis was initially discovered in macrophages; however, it was subsequently shown to be present in various immune cells. Recently, it was found that pyroptosis of immune cells was likely to promote a powerful immune response to treat cancers (23). Pyroptosis can further enhance the efficacy of immunotherapies for cancer through stimulation of the immune system with an increase in the number of both immune cells and immune factors (30). Some previous studies in melanoma cell proliferation regulation indicated that NLRP3 was associated with the IL-1β secretion and T cell response suppression in the TME (26). Moreover, other studies found that the expression of GSDMD and GSDME was related to the activation of immune cells including CD4+ T cells, CD8+ T cells, and macrophages in most solid tumors (23). Taniguchi and Karin proposed that chronic inflammation and persistent infections are closely associated with cancer onset, proliferation, aggression, and angiogenesis (55). In addition, local inflammation induced from pyroptosis was found to cause the development of local immune escape, with a possible link to carcinogenesis (56). In certain types of solid tumors, inflammatory disease was considered to be one form of precancerous lesion. This kind of chronic inflammation was correlated with the suppression of anti-tumor immune responses regulated by immune cells including CD8 T cells, NK cells, and M1 macrophages. Tumor immunity was also suppressed by specific subpopulations of immune cells recruited by the tumor cells themselves, including regulatory T cells and M2 macrophages (6). Simultaneously, in our study, PD1, PD-L1, CTLA4, LAG3, and TIGIT were significantly lower in the high-risk groups than those in the low-risk groups. The five immunosuppressor molecules were related to the efficacy of immune checkpoint inhibitors. The low expression level of immunosuppressor molecular and the decreased infiltration of tumor-infiltrating lymphocytes were considered as the characteristics of immunologically “cold” tumor, which was difficult to get benefits from ICB therapy. Consequently, we supposed that pyroptosis inducers could recruit immune cells to change the TIME, which was possible to turn immunologically “cold” tumor into “hot” tumor to improve the efficacy of ICBs for patients in the high-risk group (56).

Currently, patients with TNBC were mainly treated with chemotherapy regimens based on paclitaxel, anthracyclines, and cyclophosphamide (57, 58). Notably, neoadjuvant chemotherapy has been found to improve the treatment effect of patients in early-stage TNBC. Doxorubicin combined with carboplatin chemotherapy regimen increased pathologic complete remission (PCR) rate to 55% in patients with TNBC (59). Although the combination of carboplatin improved the PCR rate, the treatment strategy for patients with TNBC still needs further exploration. In recent years, immunotherapy and targeted therapy served significant roles in TNBC treatment. However, immunotherapeutic drugs with satisfactory efficacy in the treatment of TNBC have still not been found. Finally, we explored the predictive efficacy of the six-pyroptosis-associated gene risk models for TNBC chemotherapy sensitivity. As a result, the IC50 values were significantly higher in the low-risk group for certain anti-tumor agents, such as Lapatinib and Fulvestrant. As for Paclitaxel, Vinorelbine, Tamoxifen, and Epirubicin, the opposite results were achieved. Consequently, we proposed a potential predictive model for the sensitivity of patients with TNBC to anti-cancer drugs. Unfortunately, the lack of clinical trial validation was the major limitation of the study.

On the basis of the six pyroptosis-associated genes, we used bioinformatic methods to construct a pyroptosis-related risk model to predict the prognosis of patients with TNBC. The results of our study indicated that this risk model was an independent prognostic factor for patients with TNBC and closely associated with immune infiltration and the sensitivity of anti-cancer drugs in TNBC. However, some limitations should be noticed as well. In the current study, the research data were extracted from the METABRIC and GEO databases, which were analyzed only by bioinformatics. The validation experiments *in vivo* or *in vitro* will be further conducted to validate the predictive efficacy of the risk model. In addition, it requires more studies to investigate the molecular mechanisms of pyroptosis in TNBC. Moreover, a wider range of multi-center clinical trials is important to confirm the efficacy of combination regimen of pyroptosis inducers and immunotherapies. In conclusion, our study was the first one to establish a pyroptosis-associated gene prognostic prediction model in TNBC. These outcomes will provide new insights in the combination of pyroptosis inducers with other therapeutic strategies for TNBC.

## Conclusion

A six-pyroptosis-related gene signature was constructed by bioinformatic methods and associated algorithms, which was identified as an independent prognostic factor for patients with TNBC. Our research provides an important basis for future studies in combining proptosis with immunotherapies and chemotherapies in TNBC.

## Data availability statement

The datasets presented in this study can be found in online repositories. The names of the repository/repositories and accession number(s) can be found in the article/**Supplementary Material**.

## Author contributions

Acquisition of data (databases acquiring and data processing, etc.): PQ and QG; Analysis and interpretation of data (e.g., statistical analysis, biostatistics, computational analysis): QG, PQ, and JC; Writing, review, and/or revision of the manuscript: QG, KP, PQ, and JL; Administrative, technical, or material support (i.e., reporting or organizing data, constructing databases): PQ and QG; Study supervision: JL. All authors contributed to the article and approved the submitted version.

## Conflict of interest

The authors declare that the research was conducted in the absence of any commercial or financial relationships that could be construed as a potential conflict of interest.

## Publisher’s note

All claims expressed in this article are solely those of the authors and do not necessarily represent those of their affiliated organizations, or those of the publisher, the editors and the reviewers. Any product that may be evaluated in this article, or claim that may be made by its manufacturer, is not guaranteed or endorsed by the publisher.

## Supplementary material

Supplementary Figure 1 The outcomes of the univariate and multivariate Cox regression analysis involved significant clinical characteristic parameters associated with survival in METABRIC group. The forest plots for univariate Cox regression analysis indicate that AJCC stage (stages I vs. II vs. III) and risk score (high risk vs. low risk) were prognosis associated variables **(A)**. The forest plots for multivariate Cox regression analysis indicate that AJCC stage (stages I vs. II vs. III) and risk score (high risk vs. low risk) were independent prognostic factors **(B)**.

Supplementary Figure 2 The relevance of the ESTIMATE score and pyroptosis-associated risk model. The distributions of stromal score, immune score and ESTIMATE score were different between high- and low-risk groups in the METABRIC set **(A–C)** and GEO set **(D–F)**. The red represents the scores of patients in high-risk group, the green represents the scores of patients in low-risk group (P< 0.05).

Supplementary Figure 3 Immune infiltrations of training and validation groups. Relative proportion of immune infiltration in METABRIC **(A)** and GEO **(B)** sets.

Supplementary Figure 4 The pyroptosis-associated signature as a potential predictor for the sensitivity of anti-cancer drugs. The respective IC50 value of chosen anti-cancer drugs (Paclitaxel, Vinorelbine, Tamoxifen, Lapatinib, Epirubicin, and Fulvestrant) in relation to the risk score, as shown by Pearson’s correlation analysis, appeared to associate with the pyroptosis-related signature (|Pearson’s correlation| > 0.25 and P< 0.05).
