# Supplementary figures and images for: A pyroptosis-associated gene risk model for predicting the prognosis of triple-negative breast cancer
Source: Front Oncol. 2022 Oct 6;12:890242. doi: 10.3389/fonc.2022.890242 (PMC9582146; doi:10.3389/fonc.2022.890242)

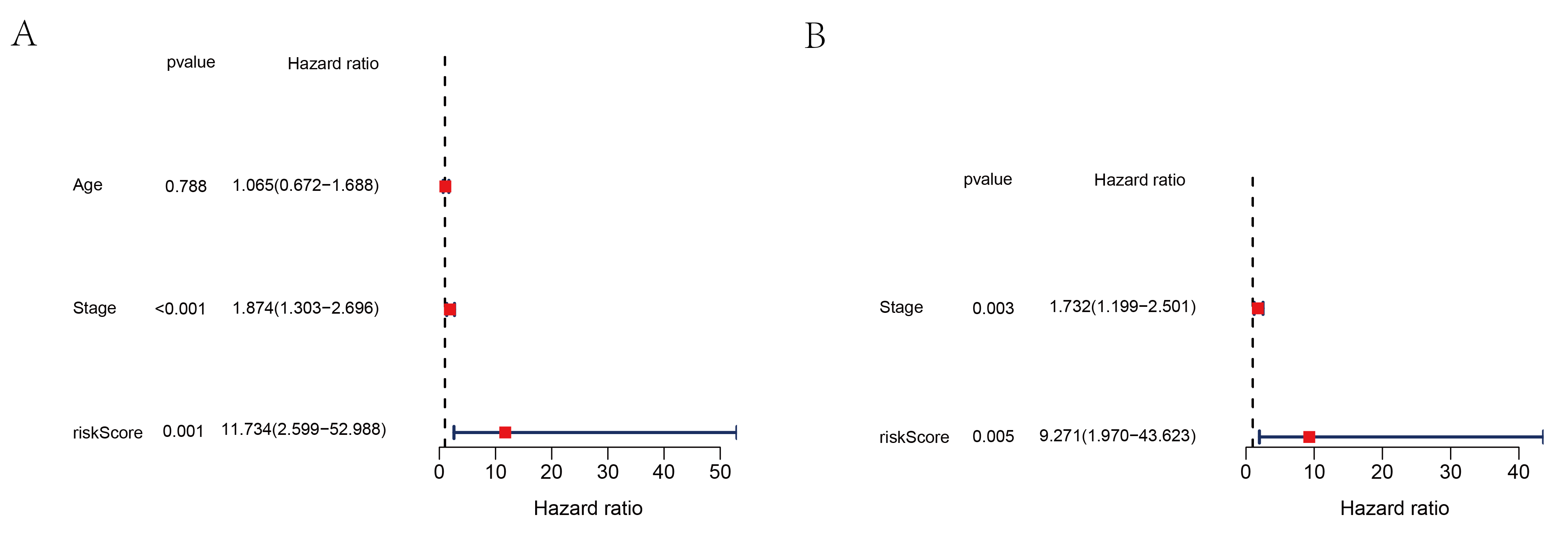

Supplement: Supplementary Figure 1 — The outcomes of the univariate and multivariate Cox regression analysis involved significant clinical characteristic parameters associated with survival in METABRIC group. The forest plots for univariate Cox regression analysis indicate that AJCC stage (stages I vs. II vs. III) and risk score (high risk vs. low risk) were prognosis associated variables (A). The forest plots for multivariate Cox regression analysis indicate that AJCC stage (stages I vs. II vs. III) and risk score (high risk vs. low risk) were independent prognostic factors (B). [file Image_1.tif]

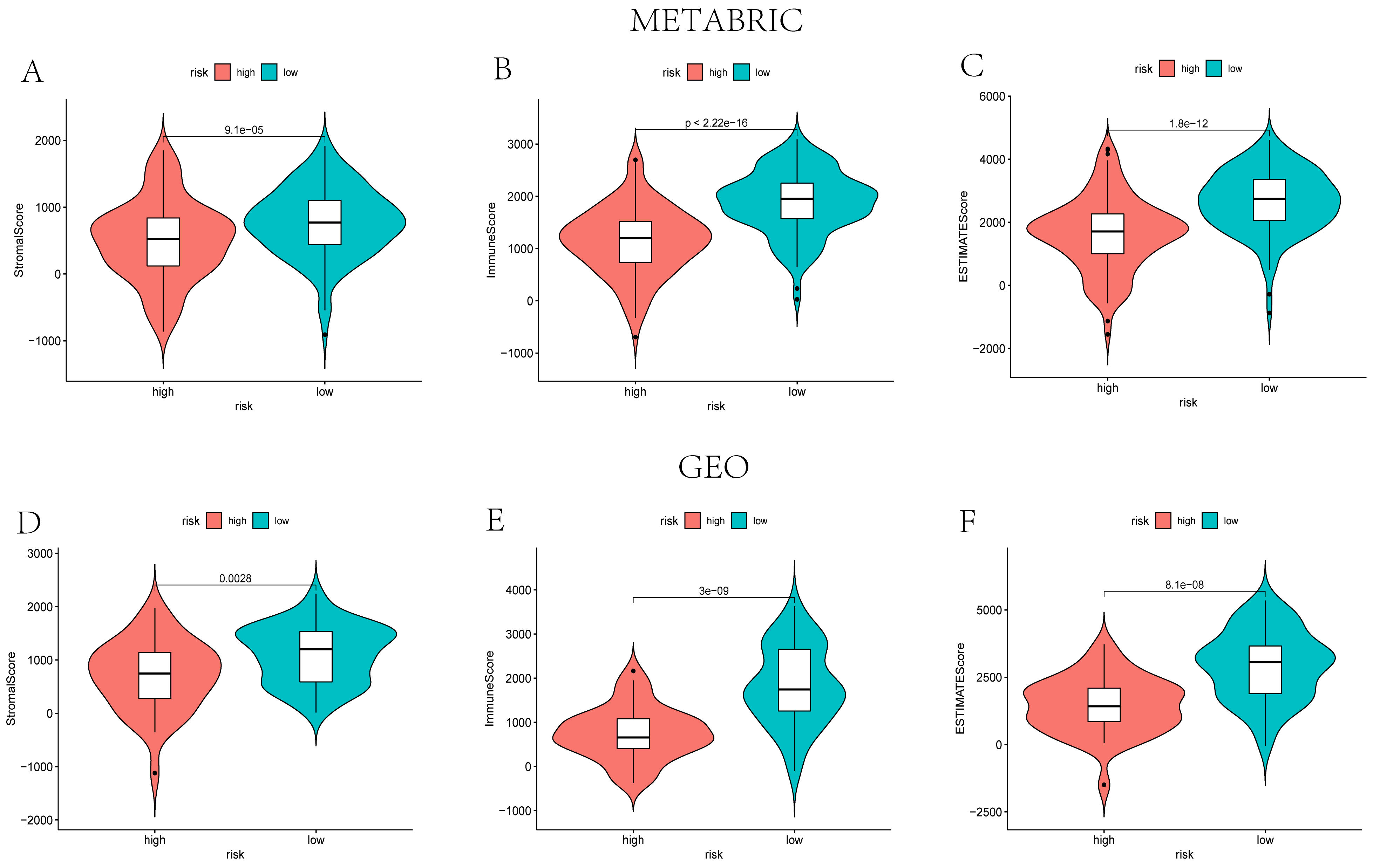

Supplement: Supplementary Figure 2 — The relevance of the ESTIMATE score and pyroptosis-associated risk model. The distributions of stromal score, immune score and ESTIMATE score were different between high- and low-risk groups in the METABRIC set (A–C) and GEO set (D–F). The red represents the scores of patients in high-risk group, the green represents the scores of patients in low-risk group (P< 0.05). [file Image_2.tif]

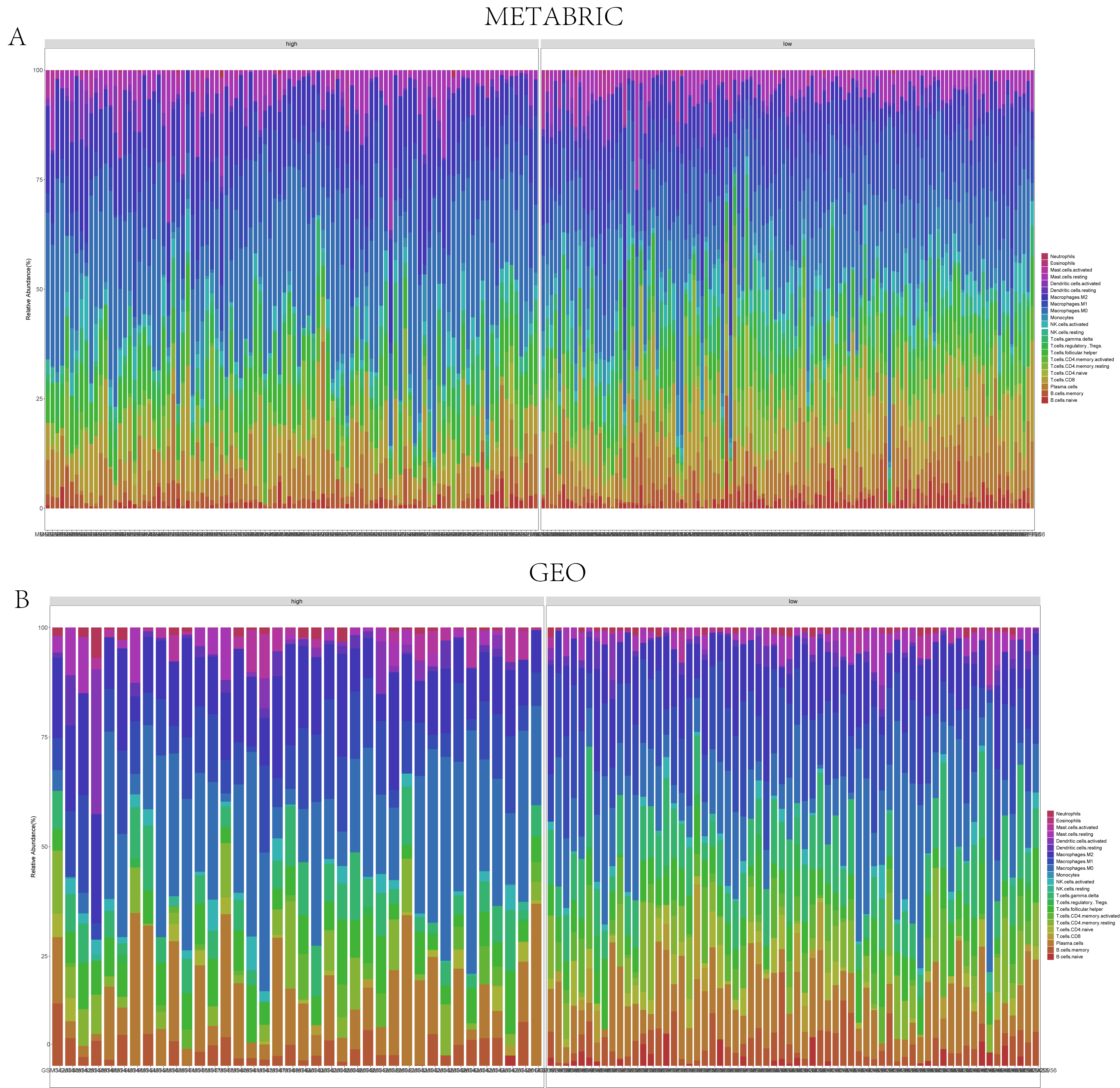

Supplement: Supplementary Figure 3 — Immune infiltrations of training and validation groups. Relative proportion of immune infiltration in METABRIC (A) and GEO (B) sets. [file Image_3.tif]

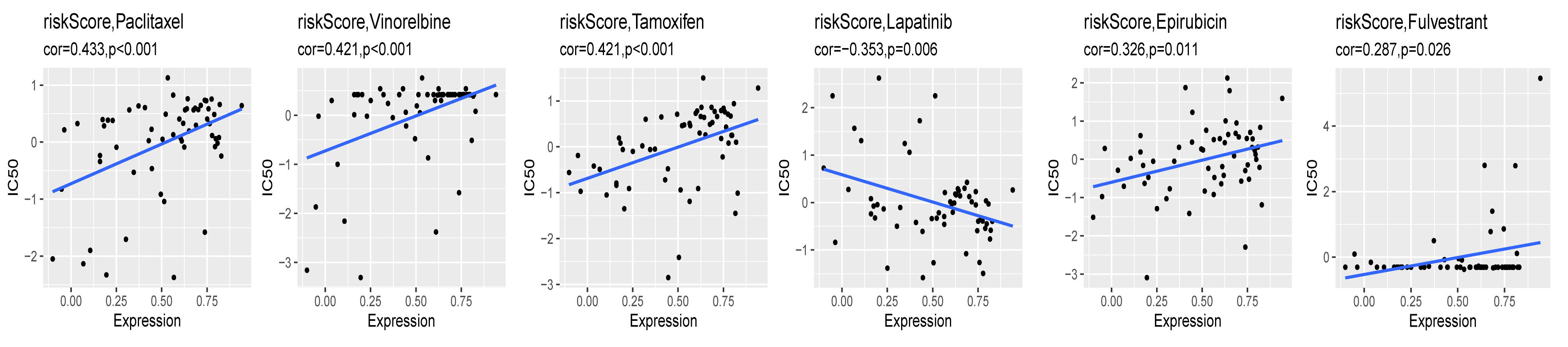

Supplement: Supplementary Figure 4 — The pyroptosis-associated signature as a potential predictor for the sensitivity of anti-cancer drugs. The respective IC50 value of chosen anti-cancer drugs (Paclitaxel, Vinorelbine, Tamoxifen, Lapatinib, Epirubicin, and Fulvestrant) in relation to the risk score, as shown by Pearson’s correlation analysis, appeared to associate with the pyroptosis-related signature (|Pearson’s correlation| > 0.25 and P< 0.05). [file Image_4.tif]

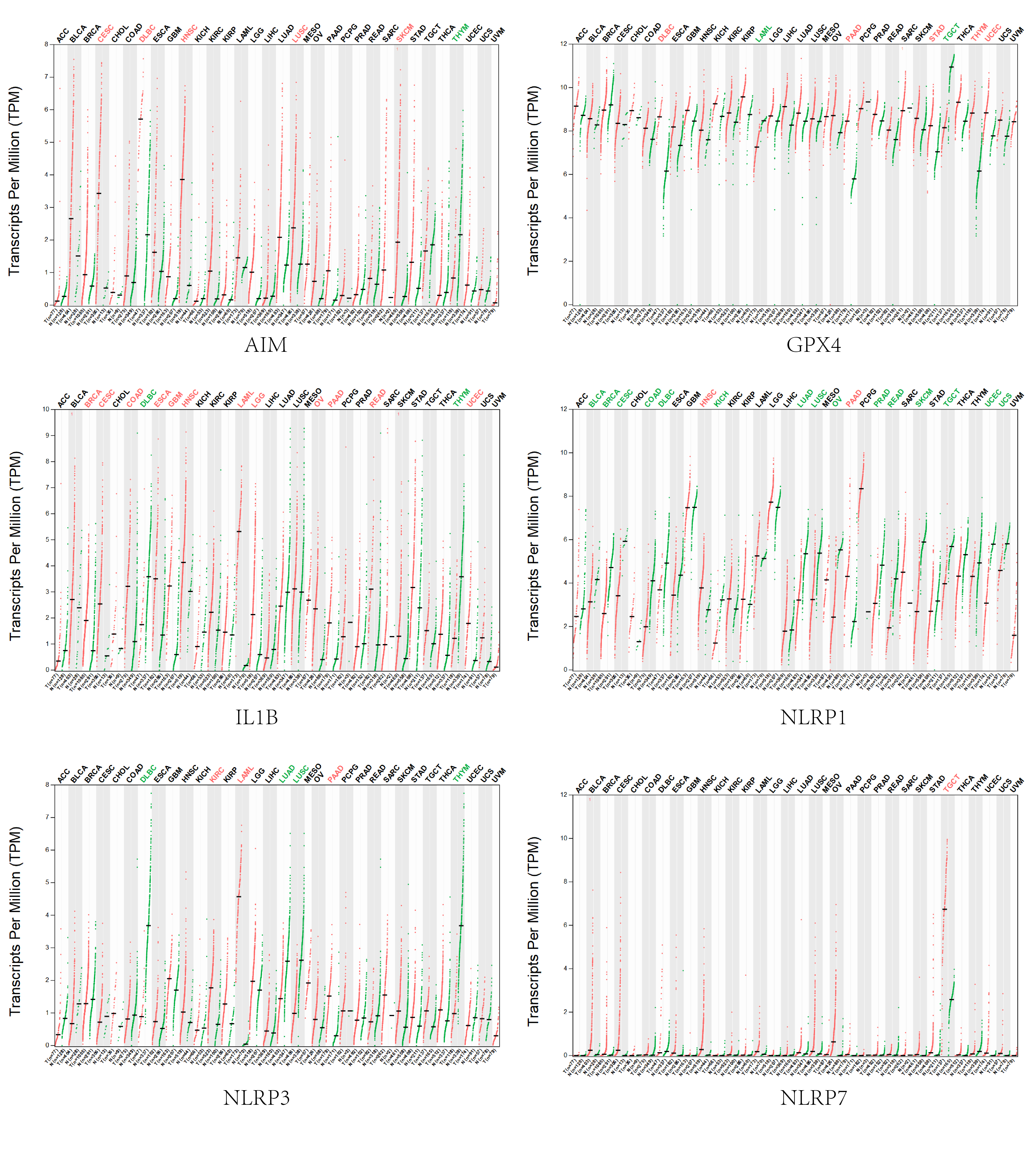

Supplement: Supplementary file 5 [file Image_5.tif]
